# NF-κB signaling in rheumatoid arthritis with focus on fibroblast-like synoviocytes

**DOI:** 10.1186/s13317-020-00135-z

**Published:** 2020-08-08

**Authors:** Leila Nejatbakhsh Samimi, Elham Farhadi, Mohammad Naghi Tahmasebi, Ahmadreza Jamshidi, Arash Sharafat Vaziri, Mahdi Mahmoudi

**Affiliations:** 1 Rheumatology Research Center, Shariati Hospital, Tehran University of Medical Sciences, Kargar Ave, Tehran, Iran; 2 Inflammation Research Center, Tehran University of Medical Sciences, Tehran, Iran; 3 Department of Orthopedics, Division of Knee Surgery, Shariati Hospital, Tehran University of Medical Sciences, Tehran, Iran

**Keywords:** NF-κB signaling, Rheumatoid arthritis, Fibroblast-like synoviocyte, Inflammation

## Abstract

The nuclear factor-κB (NF-κB) signaling pathway regulates multiple processes in innate and adaptive immune cells. This pathway is involved in inflammation through the regulation of cytokines, chemokines, and adhesion molecules expression. The NF-κB transcription factor also participates in the survival, proliferation, and differentiation of cells. Therefore, deregulated NF-κB activation contributes to the pathogenesis of inflammatory diseases. Rheumatoid arthritis (RA) is classified as a heterogeneous and complex autoimmune inflammatory disease. Although different immune and non-immune cells contribute to the RA pathogenesis, fibroblast-like synoviocytes (FLSs) play a crucial role in disease progression. These cells are altered during the disease and produce inflammatory mediators, including inflammatory cytokines and matrix metalloproteinases, which result in joint and cartilage erosion. Among different cell signaling pathways, it seems that deregulated NF-κB activation is associated with the inflammatory picture of RA. NF-κB activation can also promote the proliferation of RA-FLSs as well as the inhibition of FLS apoptosis that results in hyperplasia in RA synovium. In this review, the role of NF-κB transcription factor in immune and non-immune cells (especially FLSs) that are involved in RA pathogenesis are discussed.

## Introduction

Rheumatoid arthritis (RA) is classified as an autoimmune inflammatory disease that is characterized by chronic inflammation in synovial tissue and results in joint destruction [1]. The etiology of RA is not clearly known, but a large number of in vitro and in vivo studies have implied that fibroblast-like synoviocytes (FLSs) in the synovial intimal lining play a key role in RA pathogenesis. It has been confirmed that FLSs are directly responsible for joint damage by perpetuating inflammation and driving autoimmunity. The joint lining consists of two anatomical compartments: the intimal lining layer and the sub-lining layer. Macrophage-like synovial cells (MLSs) and FLSs are two major cell types that cover the intimal lining of the synovium. Both layers display remarkable changes in RA. Hyper-cellularity caused by the increased number of both mentioned cell types is a typical change that occurs before clinical manifestation [2]. Two-thirds of the resident synoviocytes are FLSs, which are considered the primary effectors of cartilage and bone destruction because of their inherent invasive properties [3]. In hyperplastic synovium, the loss of protective properties like lubricin secretion and changes in the protein-binding characteristics of the cartilage surface result in enhanced FLS adhesion and promoted invasion [4]. Despite the genotoxic synovial environment of RA, completed apoptosis of FLSs is rare. The reason would be related to the limited ability of tumor-suppressor gene p53, increased expression of anti-apoptotic proteins B cell lymphoma 2 (BCL-2) and myeloid cell leukemia 1 (Mcl-1), and dysregulation of signal transduction pathways that regulate FLS survival, especially nuclear factor-κB (NF-κB) pathway [5]. Many studies have indicated the importance of deregulated NF-κB activation in the pathogenesis of several autoimmune-based diseases, including RA.

NF-κB proteins constitute a family of inducible transcription factors which regulate many genes involved in different immune-inflammatory responses [6]. This family consists of NF-κB1 (p50), NF-κB2 (p52), RelA (p65), RelB, and c-Rel, which contribute to the transcription of target genes by forming different types of heterodimers. The most current heterodimers are p50/RelA, called classic NF-κB, and p50/c-Rel that binds to distinct sites of DNA (NF-κB-dependent promoters) and mediates inflammatory responses [7, 8]. NF-κB activation is regulated by two major signaling pathways, canonical and non-canonical pathways. A variety of stimuli, including cytokines, growth factors, pattern recognition receptors (PRRs), T cell receptors (TCRs), and B cell receptors (BCRs), activate the canonical pathway of NF-κB. Members of the TNF receptor superfamily (TNFSF), such as lymphotoxin-β receptor (LTβR), CD40, receptor activator of nuclear factor κ B (RANK), and B-cell activating factor receptor (BAFF-R), activate the non-canonical pathway of NF-κB [9, 10]. The inactive cytoplasmic form of NF-κB remains latent, and its translocation to the nucleus is inhibited by an inhibitory protein called IκB. The IκB kinase (Iκκ) complex consists of Iκκα, Iκκβ, and a regulatory subunit named NF-κB essential modulator (NEMO) or Iκκγ [9]. Both Iκκα and Iκκβ are able to phosphorylate IκB, which leads to IκB ubiquitination and proteasomal degradation [11]. The phosphorylation of Iκκα and its effect on p100 (a larger precursor protein of p52) phosphorylation (resulting in p52 generation) are known as essential events for NF-κB activation through the non-canonical pathway. Moreover, the NF-κB-inducing kinase (NIK) can activate the non-canonical pathway through p100 processing. The canonical pathway is activated by Iκκβ phosphorylation that results in Iκβ phosphorylation and degradation [12]. It seems that the canonical NF-κB pathway is involved in most aspects of immune responses, but the non-canonical pathway is supposed to be an alternative axis that contributes with the canonical pathway to regulate the specific functions of adaptive immune responses [9]. The NF-κB signaling pathway contributes to the regulation of many genes that are involved in inflammation, immune responses, and cell proliferation and survival [13]. Given the role of the NF-κB pathway in these processes, it is not surprising that NF-κB signaling is one of the most critical pathways in chronic inflammatory diseases.

### Synovial biology in rheumatoid arthritis

Joint inflammation, which is a result of interactions between synovial fibroblasts, immune cells, and mediators, leads to articular destruction, joint erosion, and disability. Cytokine production from different cell populations in RA synovium has a significant role in RA pathogenesis [14]. Cell populations have two types of interaction: 1. through cytokines and other secreted mediators, and 2. direct cell–cell interaction.

Among different cell populations, dendritic cells (DCs), synovial macrophages, synovial fibroblasts, and infiltrating T lymphocytes are the most common and abundant cells in RA synovium. CD4 + T-cell subsets (T helper cells) contribute crucially to RA pathogenesis by secreting a wide range of pro-inflammatory cytokines and chemokines. Furthermore, activated CD4 + T cells can stimulate synovial fibroblasts, monocytes, and macrophages to produce inflammatory cytokines such as TNF-α, IL-1, and IL-6 [15].

Studies have shown that together with other subsets of CD4 + T cells such as T helper 1 (Th1) cells, T helper 17 (Th17) cells play a crucial role in advancing synovial inflammation during RA development [16, 17]. It has been well documented that the imbalance between bone resorption and bone formation can result in bone erosion [18]. Th17 cells can mediate osteoclastogenesis through interleukin 17 (IL-17) secretion. The activation of Th17s can also result in the increased activity of B cells, macrophages, and neutrophils [19]. Furthermore, it has been shown that IL-17 can induce the production of interleukin 6 (IL-6) and interleukin 8 (IL-8) by RA-FLSs [20].

Autoantibodies like anticitrullinated protein/peptide antibodies (ACPAs) are detected before the onset of rheumatoid arthritis. The presence of pro-inflammatory mediators (like IL-8) and cellular stress in RA synovium trigger the expression of protein arginine deiminase (PAD) enzymes and citrullinated proteins by FLSs, which sensitizes FLSs to the effects of ACPAs, which can promote FLS migration [21].

Moreover, FLSs can produce several inflammatory mediators such as IL-1, 4, 6, 8, 10, 12, 13, 15, 17, 18, 21, interferon-gamma (IFN-γ), tumor necrosis factor-alpha (TNF-α), and transforming growth factor-beta (TGF-β), all of which have crucial roles in mediating inflammation [3].

Myeloid and plasmacytoid DCs, the major subtypes of dendritic cells, play pivotal roles in the initiation of joint inflammation [22]. DCs are increased in both synovial fluid and synovial tissue of RA patients compared to osteoarthritis patients [23]. These cells play an essential role in the initiation of antigen-specific T-cell responses and the establishment of inflammation. Dendritic cells also produce inflammatory cytokines such as TNFα, IL-1, and IL-6, which perpetuate rheumatoid synovitis [24].

It has been shown that B cells play important roles in RA pathogenesis. The number of autoreactive B cells in the peripheral blood of RA patients is 3.4-fold higher compared to healthy controls [25]. It has been reported that B cell depletion results in reduced joint tenderness and swelling in RA patients. However, recurrence of the symptom was seen after B cell regeneration [26, 27]. In RA synovial fluid and tissue, memory B cells spontaneously produce an increased amount of RANKL compared to memory B cells in healthy individuals [28, 29]. B cell precursors are considered as the main producer of osteoprotegerin (OPG), a soluble decoy receptor which inhibits osteoclastogenesis [30]. B cells also secrete a wide range of cytokines, including granulocyte-colony stimulating factor (G-CSF), granulocyte–macrophage colony-stimulating factor (GM-CSF), TGF-β, and IL-1, 4, 6, 7, 8, 10, and 12 [31].

#### Cell–cell interactions in RA synovium

Cell–cell contact between T cells and synovial antigen-presenting cells (APCs) has been recently considered as a potential therapeutic target for RA treatment. There is a close interaction between immune cells and resident cells of RA synovium such as FLSs.

Dendritic cells maintain and perpetuate chronic inflammation in RA synovium by presenting arthritogenic antigens to adaptive immune components. Myeloid DCs (mDCs) express interleukin 23 (IL-23), which promotes the expansion of Th17 cells. An interesting fact about mature mDCs is that the grade of inflammation increases with an increase in the number of mature mDCs [22]. In addition to playing the role of APC, dendritic cells secrete cytokines including interleukin 12 (IL-12), IL-17, IL-23, and interleukin 27 (IL-27), which induce polarized Th1 responses. Furthermore, DCs can activate FLSs (through IL-17 secretion) in order to maintain chronic inflammation in RA synovium [24].

It has been shown that FLSs are able to present antigens to T cells through a major histocompatibility complex (MHC) II-restricted mechanism and initiate T-cell responses in RA synovium. Increased expression of activation markers such as CD25 and CD69 as well as inflammatory cytokines including IFN-γ, TNF-α and IL-17 was shown in T cells which were co-cultured with FLSs [32]. Moreover, phorbol 12-myristate 13-acetate (PMA)-activated T cells (co-cultured with FLSs) could activate FLSs through the interaction between leukocyte functional antigen-1 (LFA–1/CD11aCD18) on T cells and intercellular adhesion molecule-1 (ICAM-1/CD54) on FLSs. This independent antigen interaction stimulates interleukin 1 beta (IL-1β) transcription in FLSs and results in IL-1 secretion [33]. In addition, it has been revealed that all different subsets of resting T cells are able to activate FLSs, resulting in the secretion of inflammatory mediators. Furthermore, activated FLSs showed increased expression of IL-6 and IL-8 at the messenger ribonucleic acid (mRNA) and protein levels, which is important in joint inflammation [24, 34]. RA T cells (which present features of premature senescence) highly express the CX3C chemokine receptor 1 (CX3CR1 or fractalkine (FKN) receptor) that interacts with FKN on FLSs. This interaction promotes the proliferation of FLSs and enhances T cell activation and surveillance [35, 36].

#### The role of the NF-κB pathway in immune and non-immune cells

It has been shown that NF-κB is expressed ubiquitously in almost all cells, and the dysregulation of NF-κB is correlated with the pathogenesis of different diseases such as cancer and autoimmune diseases, including RA [37].

Dendritic cells differentiation, activation, and survival are deeply connected with the NF-κB signaling pathway [38]. NF-κB activation regulates both inflammatory and anti-inflammatory responses through the activation of DCs. Canonical NF-κB activation by CD40 ligation on DCs leads to the early production of inflammatory cytokines, while non-canonical NF-κB activation induces the expression of anti-inflammatory enzyme indoleamine 2,3-dioxygenase (IDO), which promotes the suppressive function of regulatory T cells [39].

It has been shown that the non-canonical NF-κB pathway in DCs plays a role in providing co-stimulatory signals to CD4 + T cells and cross-priming of CD8 + T cells [40]. Overall, the non-canonical NF-κB pathway plays a role in both inflammatory and anti-inflammatory responses in RA synovium.

It has been reported that the NF-κB pathway is important for B-cell development, maintenance, and function [41]. IKKα in B cells is required for the germinal center formation and for producing long-lived immunoglobin titers, but not for primary antibody production [42]. Moreover, NIK promotes B-cell proliferation as well as B-cell survival by providing them with survival signals. Chiefly, the non-canonical NF-κB pathway plays a crucial role in the survival, differentiation, and antibody production in B cells and plasma cells, which perpetuate and maintain chronic inflammation in RA synovium [43, 44].

Components of the canonical NF-κB pathway, especially c-Rel and RelA, play important roles in T cell receptor (TCR) signaling and following T cell activation [45]. Deregulated NF-κB signaling can result in unwanted T cell activation, which can cause inflammatory and autoimmune responses [46]. Both c-Rel and RelA are involved in Th17 generation by inducing the retinoid-related orphan receptor (ROR) γT [47, 48]. Not only is c-Rel important for the development of Th1 cells, but it also participates in the induction of forkhead box P3 (Foxp3), which is called the regulatory T (Treg) master transcription factor [49, 50].

The non-canonical NF-κB pathway has a dual role in T cell biology. Although NIK is required for Th1 and Th17 generation, which is in favor of RA development, it has been shown that NIK is also essential for Treg cell generation, which can inhibit inflammation in RA synovium [51, 52].

**Fig. 1:**
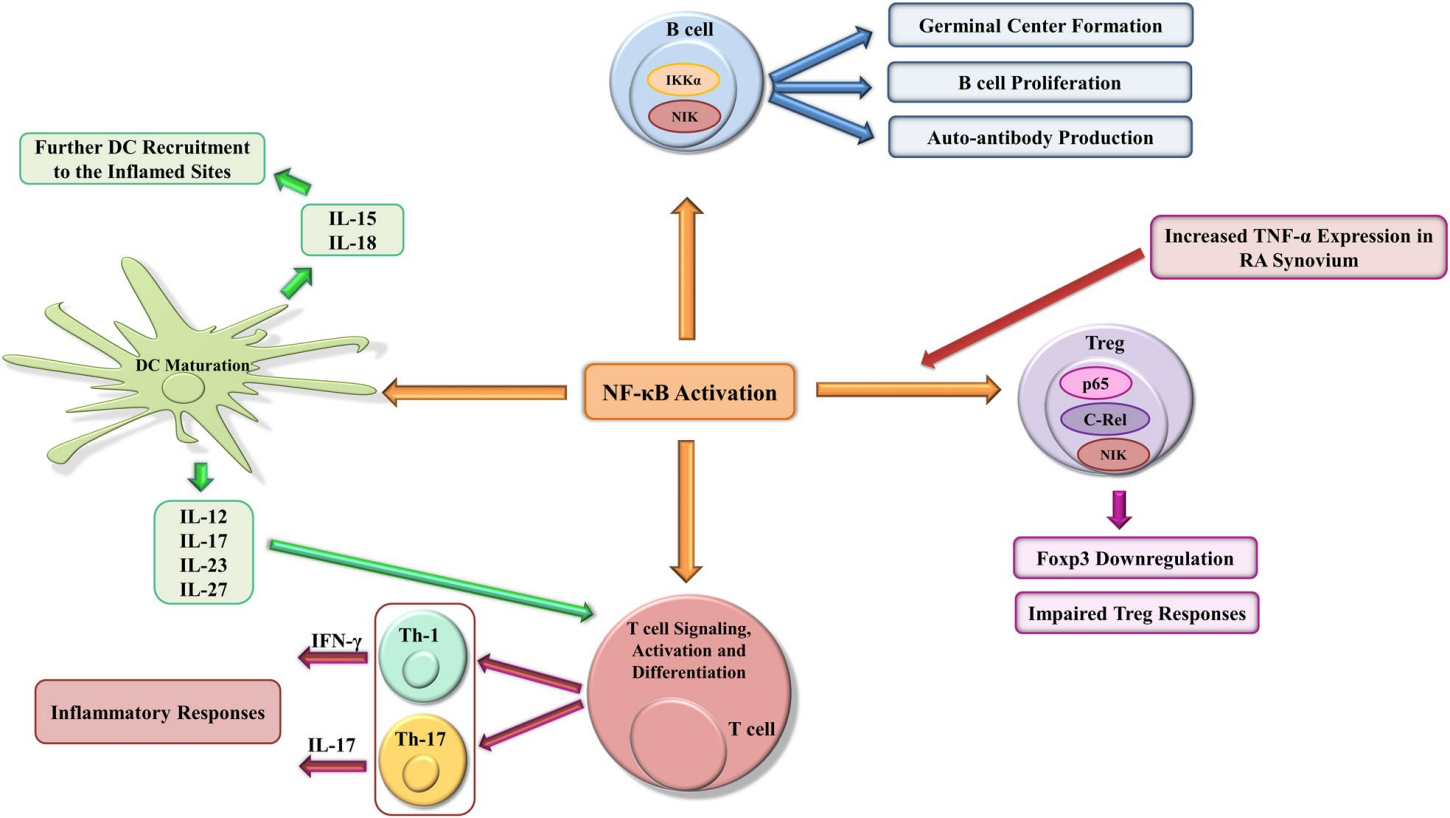
NF-κB activation perpetuates chronic inflammation by targeting genes involved in inflammation during RA development. NF-κB activation in innate and adaptive immune cells can be responsible for inflammatory responses and perpetuating chronic inflammation in RA synovium. NF-κB activation in T cells results in T cell signaling, activation, and differentiation in inflammatory T cells which produce inflammatory cytokines and maintain inflammation in rheumatoid synovium. Impaired Treg function in RA patients can be related to Foxp3 downregulation and is due to the overexpression of inflammatory cytokines such as TNF-α in the RA microenvironment. Moreover, B cell proliferation and auto-antibody production is deeply connected with activated NF-κB members. In terms of innate immune regulation, deregulated NF-κB activation in dendritic cells can cause the induction of cytokines which promote inflammatory T cell differentiation. These repeating cycles can exacerbate disease severity. NF-κB *(*nuclear factor kappa-light-chain-enhancer of activated B cells), RA (Rheumatoid arthritis), Foxp3 (Forkhead box P3), TNF-α (Tumor necrosis factor alpha)

RA synovial fibroblasts are well-known as cells that perpetuate inflammation in synovium through the secretion of pro-inflammatory cytokines and growth factors which stimulate neovascularization [2]. It had been shown that the components of the non-canonical NF-κB pathway, such as NIK, are essential for NF-κB-mediated LTβR activation in RA-FLSs [53]. RA-FLSs stimulation with the tumor necrosis factor superfamily 14 (LIGHT) leads to the upregulation of matrix metalloproteinases (MMPs) and adhesion molecules [54]. It has been reported that stimulating RA-FLSs with CD40L resulted in the increased expression of C-X-C motif chemokine 12 (CXCL12), which promotes angiogenesis, and the enhanced migration of monocytes/macrophages, B cells, and T cells to inflammatory synovium by activating the non-canonical NF-κB pathway [53]. CD40 ligation can also induce RANKL expression through NF-κB activation and enhanced osteoclast formation [55] (Fig. 1).

#### NF-κB pathway and Treg cells in RA

Regulatory T cells (Treg) play important roles in immune regulation. Different studies have reported different Treg cell counts in the peripheral blood of RA patients because of differences in the definition of CD4^+^ CD25^+^ cells. However, most studies have demonstrated that the counts of Treg cells, which are functionally impaired, were significantly increased in RA synovial fluid [56]. Tregs in RA synovial fluid fail to suppress the proliferation of effector CD4^+^ cells or the production of inflammatory cytokines such as TNF-α and IFN-γ by monocytes and CD4^+^ T cells [57].

NF-κB proteins, especially c-Rel, p65, and NIK, have important roles in the development of Tregs. It has been reported that CD4^+^ CD25^high^ cells, which express FOXP3, play a suppressive role, and mutations in FOXP3 lead to serious autoimmune disorders, such as immune dysregulation, polyendocrinopathy, enteropathy, and X-linked (IPEX) [50]. Increased TNF-α expression in RA synovium results in Foxp3 downregulation in Tregs through the TNF type II receptor. Foxp3 downregulation affects the suppressive function of Tregs to inhibit effector T cell proliferation and cytokine secretion [58]. Formation of a Foxp3-specific enhanceosome which is promoted by c-Rel and p65 is essential for the development of Tregs [50]. In vitro studies have demonstrated that c-Rel and p65 deficiency results in the blockade of Foxp3 gene expression which inhibits Treg differentiation. However, the co-expression of c-Rel and p65 leads to increased activity of the Foxp3 promoter [59]. It has been shown that c-Rel can regulate Treg differentiation indirectly through interleukin 2 (IL-2), while there is a partial defect in IL-2 production in cultured c-Rel-deficient T cells [60]. Furthermore, c-Rel is not only associated with Tregs differentiation and development, but is also required for the homeostatic proliferation of peripheral Tregs. It seems, however, that c-Rel does not affect the function of Tregs, because c-Rel-deficient Tregs can equally suppress T cell functions compared to the wild type of Tregs [61].

Several co-stimulatory molecules of the TNF receptor family which are expressed by Tregs, including tumor necrosis factor receptor 2 (TNFR2); tumor necrosis factor receptor superfamily, member 4 (TNFRSF4; CD 134, OX40); TNFRSF9 (CD137, 4-1BB); and TNFRSF18 (GITR), can activate the non-canonical NF-κB pathway through the accumulation of NIK [62]. There is controversy regarding the stimulatory or inhibitory effects of these receptors on Treg function. Although most studies have implied that the mentioned receptors suppress the function of Tregs [63–65], there are instances which indicate that these receptors enhance the number and/or suppressive function of Tregs [66–68]. It has been demonstrated that constitutive NIK expression in all T cells results in fatal multi-organ autoimmunity, which is related to the impaired suppressive function of Tregs and hyperactive effector T cell responses. A recent study showed that constitutive NIK expression leads to decreased expression of various important microRNAs and genes which are related to Treg homeostasis and its suppressive function. Furthermore, an in vivo study indicated that NIK transgenic Tregs may contribute to inflammation by losing their inhibitory function and producing inflammatory cytokines [62].

### NF-κB pathway in RA-FLSs

#### Hyperproliferation of FLSs

FLSs are considered hyperproliferative fibroblast cells with cancerous features. Several factors affect FLS mitosis and drive FLS proliferation. In vitro studies have indicated that basic fibroblast growth factor (bFGF) and platelet-derived growth factor (PDGF), which are highly expressed by FLSs, induce FLS proliferation [69]. Different cytokines such as TGF-β and activins, members of the TGF-β superfamily, are overexpressed in RA synovium and stimulate FLS proliferation [70, 71]. Moreover, mutations in the oncogene proteins and proteins involved in cell cycle regulation in RA FLSs have been documented [72–74]. Immunohistochemistry analysis has indicated the increased expression of NF-κB1 (p50) and RelA (p65) in RA synovial intimal lining cells compared to normal synovium [75]. NF-κB activation can promote the proliferation of RA-FLSs and the following hyperplasia that result in pannus formation and the consequent exacerbation of symptoms. NF-κB acts as a positive regulator of the cell cycle in fibroblasts and myoblasts by inducing the expression of cyclin D1 and c-Myc [76]. Moreover, bFGF and PDGF treatment have been shown to activate the NF-κB pathway, which results in c-Myc induction and cell proliferation. Although c-Myc has positive effects on cell growth and is overexpressed in RA synovium, it can cause cell apoptosis in the absence of survival signals that are provided by growth factors like PDGF. NF-κB activation leads to increased c-Myc expression as a stimulatory signal for cell proliferation as well as providing anti-apoptotic signals that prevent the cytotoxic effect of c-Myc in RA-FLSs. Thus, NF-κB pathway activation is involved in synovial hyperplasia in RA by inducing increased proliferation [76].

#### Decreased apoptosis

Programmed cell death (apoptosis) is a regulated cellular suicide mechanism which results in the removal of undesired cells from tissues. Although RA-FLSs express death receptors, they are relatively resistant to pro-apoptotic molecules, including TNF, Fas ligand (Fas-L), and TNF-related apoptosis-inducing ligand (TRAIL ([77]. Increased expression of proteins with anti-apoptotic effects like Bcl-2, sentrin-1, Fas-associated death domain-like IL-1 beta-converting enzyme-inhibitory protein (FLIP), Mcl-1, and protein kinase B (Akt) causes apoptosis resistance [78]. Several studies have indicated that despite frequent DNA breaking in RA synovium, the morphological signs of apoptosis are extremely rare in RA-FLSs compared to trauma or osteoarthritis (OA)-FLSs [79].

A variety of stimuli such as radiation, TNF-α, and chemotherapeutic agents can induce NF-κB activation. NF-κB activation delivers anti-apoptotic signals in different cell types by inducing the expression of anti-apoptotic genes such as the cellular inhibitor of apoptosis protein-1 (c-IAP1) and c-IAP2, tumor necrosis factor receptor-associated factor 1 (TRAF1) and TRAF2, B-cell lymphoma-extra-large (Bcl-xL), the Bcl-2 homologs A1/Bfl-1, X-linked inhibitor of apoptosis protein (XIAP), and immediate early response gene X-1 (IEX-1).

The transcriptional activity of the NF-κB-p65 subunit (which plays a crucial role in inflammatory and autoimmune diseases) is regulated by phosphorylation and acetylation. Phosphorylation of p65 Ser536 can inhibit p53 activity, resulting in FLS resistance to apoptosis [80, 81]. It has been reported that sirtuin 1 (SIRT1) is downregulated in both FLSs and RA synovium. Overexpression of SIRT1 can significantly inhibit FLS proliferation, migration, and invasiveness. SIRT1 overexpression can also suppress the NF-κB pathway by reducing p65 expression, p65 phosphorylation, and acetylation in FLSs [82]. In addition, phosphatidylinositol 3-kinase/Akt (PI3K/Akt) activation is typically detected in RA-FLSs and could potentially activate NF-κB and inhibit Fas-induced apoptosis [78]. Several studies have pointed out that overexpression of FLIP in RA synovial tissue can be involved in synovial fibroblasts survival by inhibiting Fas-mediated apoptosis. Increased expression of FLIP is directly correlated with NF-κB activation [83, 84]. Thus, NF-κB inhibition or FLIP downregulation in RA fibroblasts can promote apoptosis via the Fas-FasL pathway [85]. Generally, the NF-κB pathway, which is highly activated in RA and plays a crucial role in providing strong pro-survival and anti-apoptotic signals to FLSs, induces FLS resistance to apoptosis.

#### Cytokine production

FLSs secrete a wide range of mediators including pro-inflammatory cytokines, growth factors, MMPs, and angiogenic factors. Analyses of RA synovial tissue have indicated the high mRNA and protein expression of different inflammatory cytokines, including IL-1, IL-6, TNF-α, GM-CSF, G-CSF, and TGF-β. Among inflammatory cytokines, IL-1 and TNF-α play important roles in RA pathogenesis [86].

It is clear that the constitutive activation of the NF-κB pathway in RA is important for maintaining chronic inflammation. IkB kinase (IKK) mediates the majority of inflammatory signaling pathways. Inhibition of IKK in FLSs by IMD-0560, IκB kinase β inhibitor, results in suppression of IkBα phosphorylation that is induced by TNF-α. Therefore, IMD-0560 is able to suppress the production of inflammatory cytokines by FLSs, including monocyte chemoattractant protein-1 (MCP-1), IL-6, and IL-8 [87]. Although NF-κB proteins (p50 and p65) are detected in the nuclei of intimal synoviocytes in both RA and OA, NF-κB activation is much greater in RA than in OA due to the phosphorylation and degradation of IkBα in RA synoviocytes. In vitro treatment of FLSs with IL-1 and TNF-α leads to NF-κB signaling activation and increased cytokine production through the activation of the IKK complex. In addition, it has been demonstrated that the kinase activity of both IKKα and IKKβ is increased over tenfold within minutes of cytokine exposure [88].

Activation of IKKε, a member of the NF-κB family, in RA-FLSs of the synovial intimal lining results in JUN phosphorylation and induction of MMPs expression (independent of c-Jun N-terminal kinase (JNKs)). IKKε and serine/threonine-protein kinase TBK1 (TANK-binding kinase 1) are homologous to IKKα and IKKβ and regulate interferon-related responses in FLSs [89]. RA-FLSs can produce type I interferons, which have pro-inflammatory or anti-inflammatory roles, in response to stimulation of Toll-like receptors (TLRs) [90].

IKK2 is known as a central kinase for NF-κB activation, and the blockade of IKK2 inhibits the effects of IL-1 and TNF-α on the induction of IL-6, IL-8, and intercellular adhesion molecule-1 (ICAM-1) in FLSs [88]. It has been shown that interleukin 32 (IL-32) and IL-1 family members such as IL-18 and interleukin 33 (IL-33) are also produced by cytokine-stimulated FLSs [91].

Taken together, activated NF-κB key components in RA-FLSs contribute to pannus formation and persistent inflammation in synovial tissue through the induction of inflammatory mediators and production of destructive enzymes.

#### Invasiveness

Invasiveness is one of the prominent features of RA-FLSs. It is related to their capacity to produce inflammatory cytokines and MMPs. Cartilage erosion by FLSs develops through multiple processes which include attachment to the cartilage and synthesis of enzymes that degrade the extracellular matrix (ECM). FLSs interact with the components of cartilage ECM through the over-expression of several members of the β1 integrin family. Fibronectin-derived peptides and integrins induce the expression of MMPs [92]. It has been shown that other than integrins, ICAM-1 and particularly vascular cell adhesion molecule 1 (VCAM-1) (unique to FLSs) are overexpressed in cultured FLSs, which are able to induce MMP expression [93]. MMPs, including stromelysin-1 (MMP-3) and collagenases (MMP-1, MMP-13), play an important role in RA development. RA-FLSs secrete different types of MMPs including MMP-1, 2, 3, 8, 9, 10, 11 and 13 [86, 94–98]. Although unstimulated FLSs express MMPs at low levels, the expression of these enzymes can be induced by inflammatory cytokines including IL-1 and TNF-α and growth factors such as bFGF, PDGF, and epidermal growth factor (EGF). Moreover, IL-17 can synergistically enhance the effects of IL-1 and TNF-α and increase the expression of MMPs [99]. The expression of MMP-2, MMP-3, and MMP-9, which degrade non-collagen matrix components of the joint, is elevated in arthritis [100, 101].

**Fig. 2:**
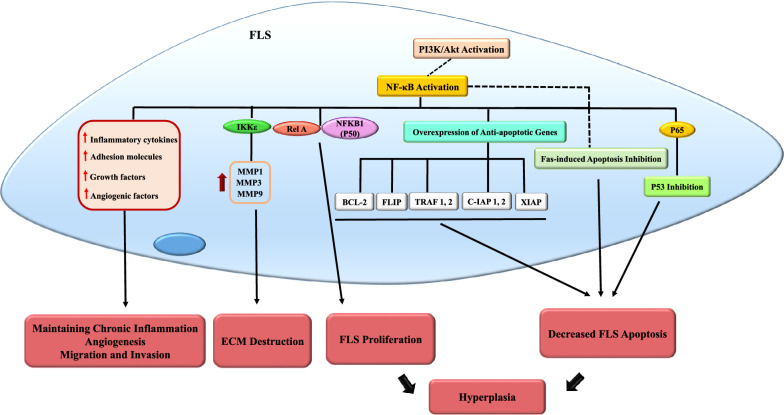
NF-κB activation in fibroblast-like synoviocytes regulate inflammatory responses in RA. Fibroblast-like synoviocytes play an important role in RA pathogenesis. NF-κB activation in FLS regulates different cell signaling processes, including decreasing FLS apoptosis by increasing the expression of anti-apoptotic genes and the inhibition of P53 and Fas as apoptosis regulatory molecules. NF-κB activation can also affect FLS proliferation and lead to FLS hyperplasia in RA synovium. Apart from this, RA-FLSs produce some growth factors which result in hyperplasia, inflammatory mediators such as inflammatory cytokines that maintain chronic inflammation in synovium, and different adhesion molecules which help further FLS migration to inflamed sites and increase their invasive characteristics. RA (Rheumatoid arthritis), NF-κB *(*nuclear factor kappa-light-chain-enhancer of activated B cells), FLS (Fibroblast-like synoviocyte), Fas (CD95)

NF-κB activation can potentially induce MMP-1, MMP-3, and MMP-9 gene expression due to the fact that the promoters of these genes have canonical binding sites for NF-κB. Although the promoter of MMP-13 does not contain an NF-κB binding site, inhibiting NF-κB signaling blocks the expression of MMP-13 (Fig. 2) [102, 103].

## Conclusion

Several lines of research have demonstrated that the pathogenesis of RA is heterogeneous, complex, and correlated with different signal transduction pathways. NF-κB and its family members are inducible transcription factors which regulate cell survival by pro- and anti-apoptotic-related gene regulation. In addition, NF-κB activation regulates various pro-inflammatory genes, such as those encoding chemokines, cytokines, and genes that are involved in inflammasome regulation. FLSs, which play a crucial role in maintaining chronic inflammation in the RA microenvironment, are hyperproliferative and invasive cells. NF-κB activation in RA-FLSs not only enhances the production of pro-inflammatory cytokines and matrix metalloproteinases, but also promotes proliferation and inhibits apoptosis, which leads to disease progression. In addition, T cell, B cell, and DCs survival, differentiation, and activation are deeply associated with NF-κB pathway activation. In immune cells, NF-κB activation is not only required for CD8 + T cells cross-priming, supplying co-stimulatory signals to CD4 + T cells and autoantibody production by B cells, but also increases the production of inflammatory cytokines and growth factors.

NF-κB members have paradoxical roles in the generation of Treg cells. Some NF-κB members, such as c-Rel, are essential for Treg development because of their participation in the formation of the Foxp3-specific enhanceosome and induction of Foxp3 expression, while deletion of the IKK-negative regulator (CYLD) or constitutive expression of active IKKβ is in favor of Treg development.
